# Human umbilical cord mesenchymal stem cell-derived microvesicles alleviate pulmonary fibrosis by inhibiting monocyte‒macrophage migration through ERK1/2 signaling-mediated suppression of CCL2 expression

**DOI:** 10.1186/s13287-025-04266-w

**Published:** 2025-03-24

**Authors:** Xiuping Liang, Yanhong Li, Yinlan Wu, Tong Wu, Deying Huang, Ziyi Tang, Lu Cheng, Chunyu Tan, Ronghui Liao, Jing Zhao, Zehui Liao, Yubin Luo, Yi Liu

**Affiliations:** 1 Department of Rheumatology & Immunology, Laboratory of Rheumatology and Immunology, West China Hospital, Sichuan University, Chengdu, Sichuan China; 2 Department of Pulmonary and Critical Care Medicine, West China Hospital, Sichuan University, Chengdu, 610041 China; 3 Meishan People’s Hospital, Meishan, Sichuan China

**Keywords:** Pulmonary fibrosis, Microvesicles derived from human umbilical cord mesenchymal stem cells, Monocyte–macrophages, CCL2/CCR2, ERK1/2

## Abstract

**Background:**

Pulmonary fibrosis (PF) is a disease with high morbidity and mortality rates, but effective treatment options are extremely limited. Mesenchymal stem cells (MSCs) and their derivatives show promise as potential therapeutics for PF. However, the underlying mechanisms responsible for these beneficial effects remain poorly understood. The objective of this study was to elucidate the specific mechanism through which microvesicles derived from human umbilical cord MSCs (MSC-MVs) alleviate PF.

**Methods:**

The effects of MSC-MVs on PF in bleomycin (BLM)-induced mice were assessed via histological staining, flow cytometry, and enzyme-linked immunosorbent assays (ELISAs). The potential therapeutic target was identified via RNA sequencing (RNA-seq) analysis, followed by validation via real-time quantitative polymerase chain reaction (RT‒qPCR), ELISAs, scratch testing, and western blotting (WB).

**Results:**

MSC-MVs significantly attenuated collagen fiber deposition and downregulated the expression of extracellular matrix components in the lungs of the BLM-induced mice. Moreover, this treatment substantially ameliorated lung inflammation by reducing the monocyte‒macrophage ratio and the TNF-α and IL-6 levels. Further analyses revealed that MSC-MVs inhibited the classic chemotactic CCL2/CCR2 axis of monocyte‒macrophages, leading to reduced recruitment of monocytes‒macrophages to the lungs, which decreased lung inflammation and prevented fibrotic progression. Both in vitro and in vivo findings demonstrated that MSC-MVs suppressed ERK1/2 phosphorylation followed by decreased CCL2 production to modulate monocyte–macrophage migration.

**Conclusions:**

Our findings demonstrate that the protective effect of MSC-MVs against BLM-induced lung toxicity was achieved through the inhibition of the ERK1/2 signaling pathway, leading to the suppression of CCL2 expression and subsequent modulation of monocyte-macrophage migration, thereby establishing a theoretical basis for the effect of MSC-MVs in PF.

**Supplementary Information:**

The online version contains supplementary material available at 10.1186/s13287-025-04266-w.

## Background

Pulmonary fibrosis (PF) is a disease involving the lung interstitium, alveoli, or bronchioles and has high morbidity and mortality rates. This disease has various etiologies, including connective tissue diseases (CTDs), viral infections, silica inhalation, prolonged smoking, radiation exposure, chemotherapy administration, radiotherapy treatment, and genetic mutations [1–3]. The pathogenesis of PF is primarily due to damage to alveolar epithelial and vascular endothelial cells by various factors [4, 5]. Subsequently, the infiltration of diverse inflammatory cells [6], fibroblast proliferation, myofibroblast accumulation, and excessive collagen deposition occur, leading to the destruction of the alveolar architecture and the development of fibrosis [7]. Consequently, regulation of the aberrant inflammatory internal environment and amelioration of abnormal cellular proliferation and extracellular matrix deposition are important strategies for treating PF. To date, very few effective treatment options are available for PF [2, 8]. Therefore, development of novel therapeutic approaches that are both safe and effective for managing PF is urgently needed.

A profibrotic inflammatory milieu is present in PF, wherein diverse immune cells and inflammatory mediators actively participate in the initiation and progression of fibrotic processes, and macrophages play a key role in these processes [9]. The lung contains various macrophage populations, including alveolar macrophages (AMs) residing in the airways and interstitial tissue-associated macrophages [10]. Resident AMs are characterized by their ability to persist within the airways and renew themselves, which allows them to respond to inflammation and injury [11]. Additionally, during lung damage or inflammation, monocytes are recruited and differentiated into macrophages to play a role in the repair process, and the number of these cells typically decreases after lung damage or inflammation has resolved [12]. However, these cells remain in PF, thereby promoting the development of fibrotic tissue [13]. Thus, targeting this population of cells may be a strategy to mitigate worsening fibrosis in PF.

Mesenchymal stem cells (MSCs) are a type of nonhematopoietic multipotent stem cell derived from the mesoderm and are widely distributed in connective tissues and organ interstitium; these cells can self-renew and differentiate into mesodermal tissues and have anti-inflammatory, immunosuppressive, antiapoptotic, antifibrotic, and proangiogenic properties [14, 15]. MSCs can exert their therapeutic effects indirectly through the secretion of extracellular vesicles (EVs), such as exosomes (EXOs) and microvesicles (MVs) [16, 17]. MSC-derived EVs serve as natural and effective delivery vehicles, maintaining functional characteristics similar to those of their parent cells [16, 18]. Allaura et al. [19] reported that MSC-EVs ameliorate the pathogenesis of Alzheimer’s disease. Zhang et al. [20] revealed that MSC-EVs alleviated arthritic progression in rheumatoid arthritis patients by restoring the macrophage balance. Numerous studies have demonstrated the ability of MSC-EVs to impede the progression of PF by safeguarding alveolar epithelial cells, inhibiting epithelial-mesenchymal transition, restraining the proliferation and differentiation of fibroblasts into myofibroblasts, and diminishing collagen deposition [21–23]. The potential of MSC-EVs to modulate the function of inflammatory cells and improve fibrotic outcomes remains unclear. In this study, we employed a mouse model of PF induced by bleomycin (BLM) to explore the therapeutic and immunomodulatory potential of MSC-MVs in the treatment of PF and the underlying mechanisms involved.

## Methods

### Statement

The work has been reported in line with the ARRIVE guidelines 2.0.

### MSC culture and MSC-MV isolation

MSCs at passage 4 (P4) were provided by the Chengdu Stem Cell Biobank, specifically obtained from a healthy donor (no.2020508), and the MSCs were cultured in complete culture media (patent number: CN202010735896.3) supplemented with 10% fetal bovine serum (FBS; Gibco, USA). When more than 80% of the P5 MSCs had proliferated and fused, the cell supernatant was discarded, and the cells were rinsed three times with phosphate-buffered saline (PBS). Then, serum-free medium was added. Next, the MSCs were starved for 48 h in a 5% CO2 incubator at 37 °C to induce MVs release.

The supernatants of the above cells were collected and centrifuged at 400 × g at 4 °C for 10 min and then centrifuged at 2000 × g at 4 °C for 20 min to remove the precipitate, which contained cell debris. The supernatant was subsequently transferred to a unique tube for ultracentrifugation (Avanti J-26 S XP, Beckman Coulter, Brea, CA, USA) and centrifuged at 50,000 × g at 4 °C for 2 h to obtain a pellet, which was subsequently resuspended in PBS, aliquoted, and stored at − 80 °C for further study. MVs were thawed and diluted to the correct concentration on the day of administration [24].

### Transmission electron microscopy (TEM)

The MSC-MV suspension was dropped onto a copper grid with a carbon film for 3–5 min via a pipet gun, and excess liquid was removed with filter paper. Next, 2% phosphotungstic acid (Servicebio, CA) was added to the copper grid to stain for 1–2 min, after which the excess liquid was removed. Finally, after drying at room temperature, the cuprum grids were observed via TEM, and images were acquired (Hitachi, HT7800/HT770, OR JPN).

### Nanoparticle tracking analysis (NTA)

The size and particle concentration of the MSC-MVs were determined via a ZetaView (Particle Metrix, GER) instrument equipped with a Viton sample room and a laser (488 nm). MSC-MV suspensions were diluted with ultrapure water 500–2000 times to adjust the on-machine concentration of the sample to between 10^7^ and 10^9^ particles/ml, followed by injection into the sample room. The size and particle concentration were analyzed via ZetaView software.

### Western blotting (WB)

Total protein was extracted from the MSC-MVs, MHS cells and lung tissues of the animals via cell lysis buffer (Beyotime, CA) supplemented with protease inhibitor (1:100, Beyotime, CA) and phosphatase inhibitor (1:100, Beyotime, CA). The protein concentration was measured with a BCA protein assay kit (Beyotime, CA). Next, equal amounts of protein were heated for 5 min at 95 °C with a quarter volume of SDS–PAGE sample loading buffer (Bio-Rad, USA). Then, 25 µg of protein was separated on 10% polyacrylamide gels (EpiZyme Biotechnology CA) and transferred to polyvinylidene difluoride membranes via a Trans-Blot Mini Cell (Bio-Rad, USA). The membrane was blocked with 5% (w/v) bovine serum albumin (BSA, Servicebio, CA) for 2 h followed by an overnight incubation at 4 °C with the following primary antibodies (More information of all antibodies were showed in Supplementary Table 2): anti-CD9 (1:2000, abcam, USA), anti-CD63 (1:2000, ABSIN, CA), anti-TSG101 (1:2000, Servicebio, CA), anti-p-ERK1/2 (1:2000, Cell Signaling, USA), anti-ERK1/2 (1:2000, Cell Signaling, USA), anti-Collagen I(1:2000, abcam, USA), anti-α-SMA(1:2000, abcam, USA), anti-FN(1:5000, abcam, USA), and anti-GAPDH (1:2000, OriGene, USA). The membranes were subsequently incubated for 2 h with secondary antibody (1:10000, ZEN-BioScience, CA) at room temperature. Finally, the bands were detected and analyzed via Image Lab software.

### Animals and experimental groups

Seven-week-old male C57BL/6 mice (approximately 21–23 g) were purchased from Chengdu Enswell Biological Company and maintained for 7 days for adaptation. Throughout the experiments, the mice were maintained at 22 °C with a 12-h/12-h light/dark cycle and free access to a standard pellet diet and water. The animal experiments were approved by the Animal Ethics Committee of West China Hospital of Sichuan University (20220107007). Thirty male mice were randomly divided into 3 groups using the random number table method: the Control group, Model group, and MV group. A midline incision was made in the neck skin of all the mice to expose the trachea, and BLM (3 mg/kg; Sigma, USA) was injected into the trachea of the mice in the model and MV groups under isoflurane anesthesia, while an equivalent volume of saline was injected into the trachea of the control group mice. The mice in the MV group were then given 2*10^9 particles MSC-MVs via tail vein injection on day 2 of modeling, whereas the mice in the model group were given the same volume of PBS. Five mice from each group were sacrificed by cervical dislocation under anesthesia with isoflurane(RWD, China), after which their lungs and blood were collected at 7 days and 21 days after modeling. In the experiments of the supplementary materials, the total number of animals used reached 57, and the number of animals in each group was 4 to 5.

### Histological examinations

Lung tissues were dissected immediately after the mice were euthanized and fixed in 10% buffered formalin for 24 h. After dehydration and embedment, the tissues were sectioned at 5 μm and stained with a hematoxylin and eosin (HE) kit (Servicebio, CA) and Masson’s Trichrome Stain Kit (Servicebio, CA).

For immunohistochemical analysis, 5 μm sections were deparaffinized, rehydrated, and placed in Tris-buffered saline (TBS) supplemented with Tween-20 (Servicebio, CA). Endogenous peroxidase was blocked with 3% hydrogen peroxide (Angergech, CA), and the slides were treated with 3% BSA (Servicebio, CA) for 30 min at room temperature. The slides were incubated with primary antibody, rinsed, and incubated with an HRP-conjugated secondary antibody. Diaminobenzidine (DBA) was used to identify the reaction, followed by washing, counterstaining with hematoxylin, dehydration, clearing, and mounting with resinous mounting media. The primary antibodies used included anti-collagen I (Abcam, UK), anti-α-SMA (Abcam, UK), anti-fibronectin (FN) (Abcam, UK), anti-C-C motif chemokine receptor 2 (CCR2) (Servicebio, CA), and anti-C-C motif chemokine ligand 2 (CCL2) (Servicebio, CA) antibodies.

The remaining slides were subjected to immunofluorescence (IF). After deparaffinization, rehydration and antigen retrieval, the tissue was sealed with a PAP Pen Liquid Blocker after the section had slightly dried, and 3% BSA was added to block the tissue for 30 min. Next, the prepared primary antibody anti-F4/80 (Servicebio, CA) was added, and the slides were placed in a wet box and incubated at 4 °C overnight. Furthermore, after 3 washes with PBS for 5 min, the secondary antibody, iFluor 488-conjugated goat anti-rabbit IgG (H + L) (Servicebio, CA), was added, and the mixture was incubated at room temperature for 50 min in the dark. Then, the corresponding TSA was added, followed by microwave treatment and 3% BSA blocking. Furthermore, the secondary antibodies anti-iNOS (Servicebio, CA) and CY3 goat anti-rabbit IgG (H + L) (Servicebio, CA) were added in sequence as described above. Next, the cells were incubated with WGA working solution (Sigma, USA) in a lucifugal incubator at 37 °C for 1 h. After 3 washes, DAPI solution (Servicebio, CA) was added to the sections, which were subsequently incubated at room temperature for 10 min in the dark. Finally, after washing, autofluorescence quencher B solution (Servicebio, CA) was added for 5 min, and the samples were rinsed with running water for 10 min and coverslipped with antifade mounting medium.

### RNA sequencing (RNA-seq) and bioinformatics analysis

Lung tissues were obtained from the mice 7 days after modeling. Total RNA was extracted from the lung tissue via TRIzol^®^ Reagent (Invitrogen, USA), and genomic DNA was removed via DNase I (TaKaRa, Jan). RNA degradation and contamination were monitored on 1% agarose gels. The RNA quality was subsequently determined via a 2100 Bioanalyzer system (Agilent Technologies, USA) and quantified via an ND-2000 (NanoDrop Technologies, USA).

RNA purification, reverse transcription, library construction and sequencing were performed (Illumina, San Diego, CA). The transcriptomic library was prepared following the instructions of the TruSeq TM RNA Sample Preparation Kit from Illumina (San Diego, CA) using 1 µg of total RNA. Briefly, messenger RNA was first isolated by oligo(dT) beads according to the poly(A) selection method and then fragmented by fragmentation buffer. Second, double-stranded cDNA was synthesized via a SuperScript double-stranded cDNA synthesis kit (Invitrogen, CA) with random hexamer primers (Illumina). The synthesized cDNA was subsequently subjected to end repair, phosphorylation and ‘A’ base addition according to Illumina’s library construction protocol. Libraries were size selected for cDNA target fragments of 300 bp on 2% low-range Ultra agarose, followed by PCR amplification via Phusion DNA polymerase (NEB) for 15 PCR cycles. After quantification by TBS380, the paired-end RNA-seq library was sequenced with an Illumina NovaSeq 6000 sequencer (2 × 150 bp read length).

The raw paired-end reads were trimmed and quality controlled by fastp (https://github.com/OpenGene/fastp) with default parameters. The clean reads were subsequently separately aligned to the reference genome in orientation mode via HISAT2 (http://ccb.jhu.edu/software/hisat2/index.shtml) software. The mapped reads of each sample were assembled via StringTie (https://ccb.jhu.edu/software/stringtie/) via a reference-based approach. The thresholds for identifying significantly differentially expressed genes were an adjusted *p* value < 0.05 and a fold change > 2 or < 0.5. Gene Ontology (GO) terms associated with the biological process (BP), cellular component (CC), and molecular function (MF) categories were analyzed via a database (Goatools; https://github.com/tanghaibao/GOatools).

### Flow cytometry

The peripheral blood and bronchoalveolar lavage fluid (BALF) of all the mice at D7 were collected and centrifuged at 1200 rpm for 5 min. The supernatant was removed, and 1 ml of red blood cell lysis buffer (Leagene, CA) was added for 5 min. Then, 10 ml of PBS with 1% BSA was added, and the mixture was centrifuged at 1200 rpm for another 5 min. After centrifugation, the supernatant was removed, and 1 ml of PBS with 1% BSA was added to suspend the cells. The mixture was mixed well via a pipette, and the total number of cells was counted via an automated cell counter (JIMBIO, CA). The total cell count of each sample was recorded, and the cell suspension was transferred to flow tubes. Centrifugation at 1200 rpm was continued for another 5 min, after which the supernatant was removed. The primary antibodies were diluted in PBS with 1% BSA, and 100 µl of the mixture was added to each sample tube. Single-stain tubes were prepared by mixing 100 µl of 1% BSA in PBS with the appropriate antibody storage solution; 100 µl of PBS with 1% BSA was added to the blank tubes. The samples were incubated in the dark at 4 °C for 30 min. Next, the mixture was centrifuged at 1200 rpm for 5 min, the supernatant was discarded, and 200 µl of PBS with 1% BSA was added to resuspend the cells before proceeding with machine detection. The primary antibodies included anti-Ly6G (PerCP-Cy 5.5, BD, USA), anti-CD11b (FITC, BD, USA), anti-Ly6C (PE-Cy7, BD, USA), anti-F4/80 (BV421, BD, USA), anti-CD206 (APC, BD, USA), anti-iNOS (PE, eBioscience, USA), and anti-CCR2 (PE, BioLegend, USA) antibodies (More information of all antibodies were showed in Supplementary Table 3).

### Cell culture and treatment

The murine AM line MHS and the mouse monocyte/macrophage line RAW264.7 were purchased from the Meisen Chinese Tissue Culture Collection (CTCC, Manassas CA) and cultured in RPMI 1640 medium (Gibco, USA) supplemented with 10% FBS (Gibco, USA).

When MHS cells were cultured to 80–90% confluence in twelve-well plates, 1*10^8 particles / mL MVs were added, followed by the addition of 100 ng/ml lipopolysaccharide (LPS, Sigma, USA) after 1 h.

### MV labeling and uptake

PKH26 (Sigma, USA) was used to label the MSC-MVs (a 1:1000 dilution of commercial stock), which were then incubated for 0.5 h, washed with 3 ml of complete medium, and centrifuged at 50,000 × g for 2 h. Then, the MSC-MVs were resuspended in 50 µl and kept in the dark. After they were cultured to 80–90% confluence, MHS cells were trypsinized and resuspended in 500 µl of CFSE (Thermo Fisher Scientific, USA) dye liquor (a 1:2000 dilution of commercial stock). Then, 1 × 105 cells were cultured in confocal dishes. After culture to 80–90% confluence, the MHS cells were cocultured with PKH26-labeled MSC-MVs for 2 h, and the distribution and intensity of fluorescence were observed via fluorescence microscopy.

### Real-time quantitative polymerase chain reaction (RT‒qPCR)

Total RNA was isolated via a FastPure Cell/Tissue Total RNA Isolation Kit (Vazyme, CA), and cDNA was synthesized via HiScript III RT SuperMix for qPCR (+ gDNA wiper) (Vazyme, CA). Quantitative PCR was performed via a Bio-Rad iCycler iQ6 system (Bio-Rad, Hercules, CA) with ChamQ SYBR qPCR Master Mix (Vazyme, CA). Relative changes in expression were determined via normalization to the expression of GAPDH (Ct value). The comparative threshold (ΔΔCt) was calculated between different experimental conditions. The sequences of the primers used for CCL2 and GAPDH are listed in Supplementary Table 1.

### Scratch assay

The MHS cell supernatants were collected after coculture with MVs and/or LPS. When the RAW264.7 cells were cultured to 80–90% confluence in a twelve-well plate with a 500 μm linear gap created by scratching the cells via a SPLScar Scratcher (SPL Life Science, Korea), the MHS cell supernatants from the three groups were added. After 6 h, the width of the gaps was measured, and the relative migration rates (%, 1 - the width of the gap/500 µm) of the raw material in the three groups were calculated.

### Statistical analysis

Data with unequal sample sizes were analyzed via one-way ANOVA followed by the Sidak multiple comparison test (GraphPad Prism 8.0.1), whereas for non-normally distributed data, differences between two groups were determined via the Mann‒Whitney U test for unpaired observations. The Shapiro-Wilk test was used to check whether the data is normally distributed. Variables are reported as the mean ± SEM. A *p* value of < 0.05 was considered to indicate statistical significance.

## Results

### MSC-MVs ameliorate BLM-induced PF

MSC-MVs were isolated and identified via TEM, NTA, and WB analysis. MSC-MVs exhibited a typical cup-shaped morphology (Supplementary Fig. 1a) and expressed the CD9, CD63 and TSG101 proteins (Supplementary Fig. 1b). The mean diameter was 165.8 ± 3.0 nm (Supplementary Fig. 1c).

**Fig. 1:**
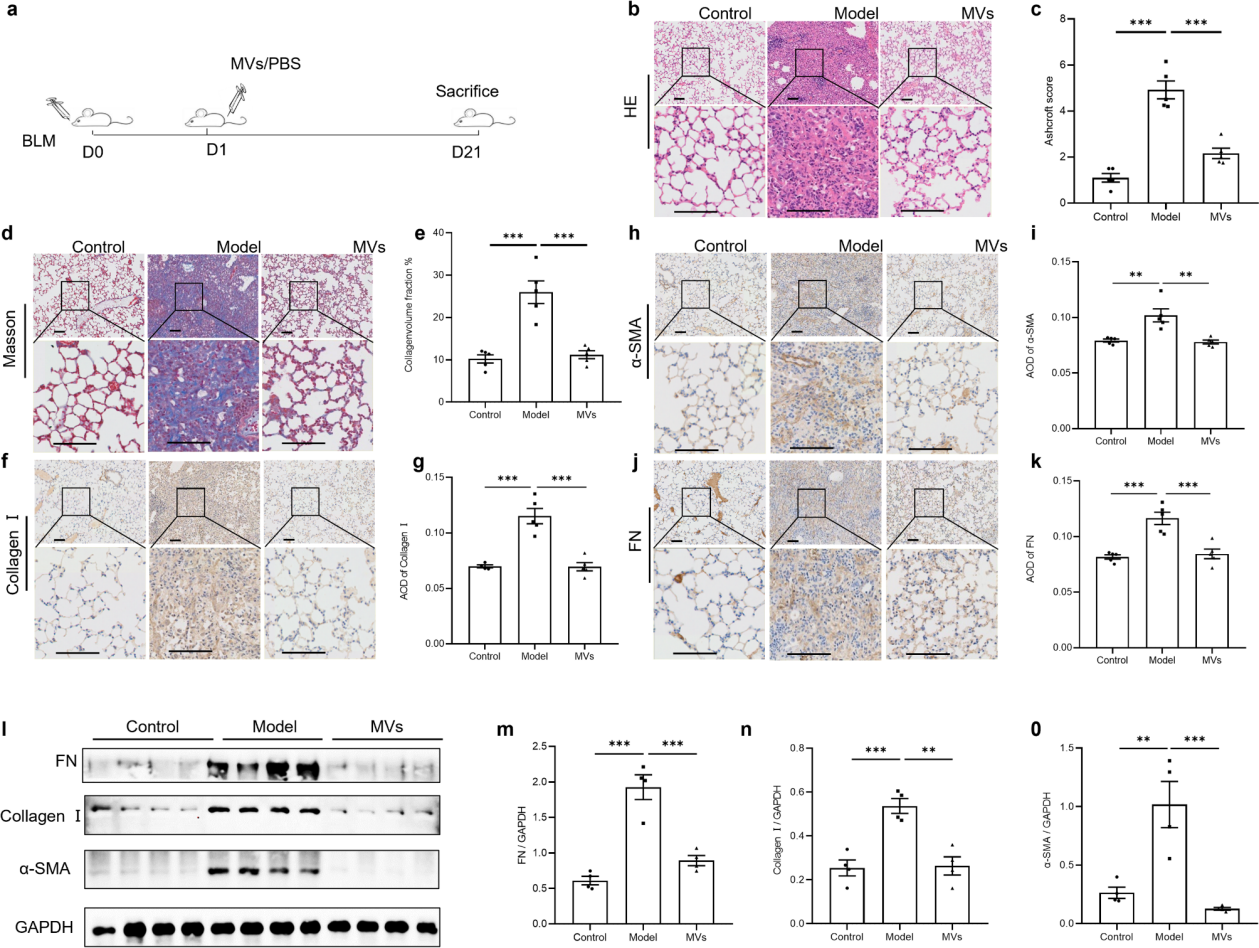
Effects of MSC-MVs on BLM-induced PF in mice. (**a**) Diagram of the experimental scheme. (**b**) HE staining of mouse lung tissue from the control, model, and MV groups. (**c**) Ashcroft score of three groups (*n* = 5). (**d**-**e**) Masson staining of mouse lung tissues from the three groups of mice and the statistical analysis of collagen-volume fraction %(*n* = 5). (**f**-**k**) Immunohistochemical staining of collagen I, α-SMA and FN and comparative analysis of the average optical density (AOD) were performed to assess positive staining in lung tissue (*n* = 5). (**l**-**o**) The corresponding levels of FN, collagen I and α-SMA in the lung tissues of mice in the control, model, and MV groups were determined by WB, with GAPDH as an internal reference The corresponding uncropped full-length blots are included in Supplementary Fig. 2d (*n* = 4). The data are shown as the means ± SEMs. The Shapiro-Wilk test of the data > 0.05. One-way ANOVA followed by the Sidak multiple comparison test was used to identify differences among the three groups, and ∗ indicates the difference between the control and model groups or the difference between the model group and the MV group. ∗∗*p* < 0.01. *** *p* < 0.001. Scale bar, 100 μm. Abbreviations: SEM: standard error of the mean

A BLM-induced PF mouse model was established to assess the effect of MSC-MVs in vivo on day 21 (Fig. 1). The results of HE and Masson’s trichrome staining revealed that, compared with that in the control group, more inflammatory cell infiltration was observed in the lung tissue of the BLM model group, along with a large increase in the number of collagen fibers and an increase in the degree of PF. However, after the injection of MSC-MVs, the number and lesion area of inflammatory cells and collagen fibers were reduced, and PF was alleviated (Fig. 1b-e). The immunohistochemical results revealed that the protein expression of collagen I, α-SMA and FN was significantly greater in the model group than in the control group and was significantly lower in the MSC-MV group than in the model group (Fig. 1f-k), and the consistent results were showed by WB (Figure l-o). These findings indicate that the administration of MSC-MVs can ameliorate BLM-induced fibrosis in mice. Meantime, MSC-MVs also show consistent therapeutic effects in female BLM mice (Supplementary Fig. 2). What’s more, the HE and Masson’s trichrome staining results of pre-experiment in vivo suggested the effects of MSC-MVs on PF exhibit a dose-dependent manner (Supplementary Fig. 3). Further, the result of administrating single-dose MSC-MVs on day 1 or day 3 or day7 post-BLM suggested that MSC-MVs had a more favorable impact in the early intervention of BLM-induced lung lesions (Supplementary Fig. 4).

### Transcriptomic response at the global level and signatures mediated by MSC-MVs in the early stages of PF induced by BLM in mice

**Fig. 2:**
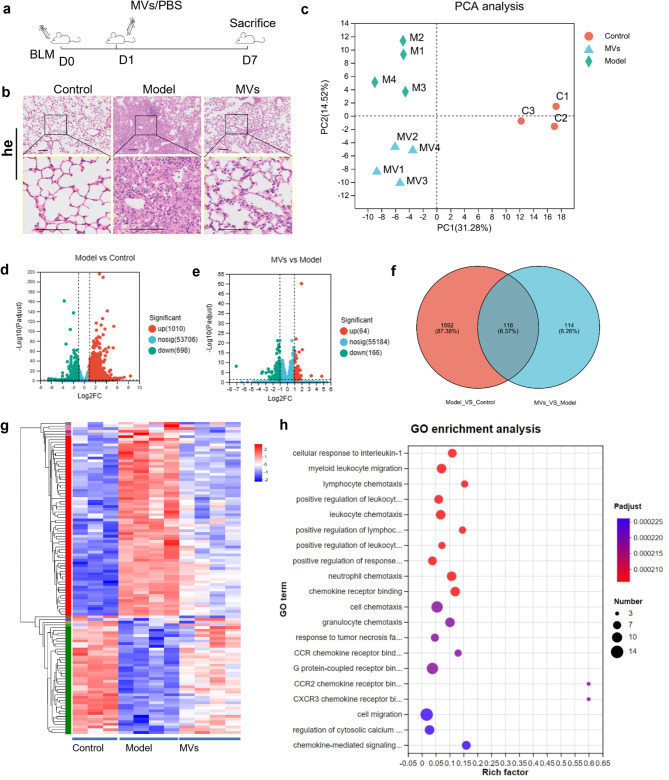
Analysis of transcriptomic changes in BLM-induced PF and MSC-MV treatment groups at D7. (**a**) Diagram of the experimental scheme. (**b**) HE staining of mouse lung tissues from the control, model, and MV groups. (**c**) PCA of transcriptomic differences among the control, model, and MV groups. The colors and shapes indicate different groups. (**d**) Volcano plot showing the changes in lung genes between the control and model groups (fold change ≥ 2). (**e**) Volcano plot showing the changes in lung gene expression between the model and MV groups (fold change ≥ 2). (**f**) Venn analysis of different genes in both the control versus model comparison and the model versus MV group comparison. (**g**) Heatmap across different groups for 116 different genes. The heatmap colors indicate normalized gene expression ranging from high (red) to low (blue) (The heatmap with gene note was showed in Supplementary Fig. 7). (**h**) Bubble chart of the GO enrichment of 116 differentially expressed genes. The color of the bubbles indicates the *p* value, and the size of the bubbles indicates the number of genes associated with the GO term

In the previous section of this study, we showed that the administration of MSC-MVs via tail vein injection can ameliorate pathological manifestations in lung tissue. We elucidated the underlying mechanism through which MSC-MVs exert their therapeutic effects on PF during the inflammatory phase. In early BLM-induced PF in mice (at 7 days after model establishment) (Fig. 2a), HE staining of lung tissue revealed that, compared with those in the model group, the number and lesion area of inflammatory cells in the MSC-MV group were substantially lower, suggesting that MVs have superior anti-inflammatory effects (Fig. 2b). Furthermore, to reveal the mechanisms of MV-mediated anti-inflammatory effects in early BLM-induced PF in mice, we performed transcriptomic profiling of lung tissue from the three groups via RNA-seq. Principal coordinate analysis (PCA) revealed that the samples in each group exhibited good clustering, and the groups were clearly separated, suggesting that the samples within each group exhibited good reproducibility (Fig. 2c). As shown in the volcano plot results in Fig. 2d, when an adjusted *p* value < 0.05 and a fold change ≥ 2 were used as the screening criterion, 698 genes were significantly downregulated and 1010 genes were significantly upregulated in the model group compared with the control group. Compared with those in the model group, 116 genes were significantly downregulated, and 64 genes were significantly upregulated in the MV group (Fig. 2e). By Venn analysis, we identified 116 distinct genes that were significantly differentially expressed between the control group and the model group and between the model group and the MV group (Fig. 2f). Moreover, a heatmap indicated that these 116 distinct genes in the model group were significantly different from those in the control group, while MV treatment seemed to reverse these changes (Fig. 2g). GO functional enrichment analysis of 116 distinct genes revealed that these genes mainly participate in immune response-related functions, such as “positive regulation of leukocyte chemotaxis”, “myeloid leukocyte migration” and “leukocyte migration” (Fig. 2h). These findings suggest that MSC-MVs regulate immune cells, indicating that the use of MSC-MVs may be a promising therapeutic for treating PF.

### MSC-MVs inhibited the migration of monocytes and macrophages in early BLM-induced PF in mice

**Fig. 3:**
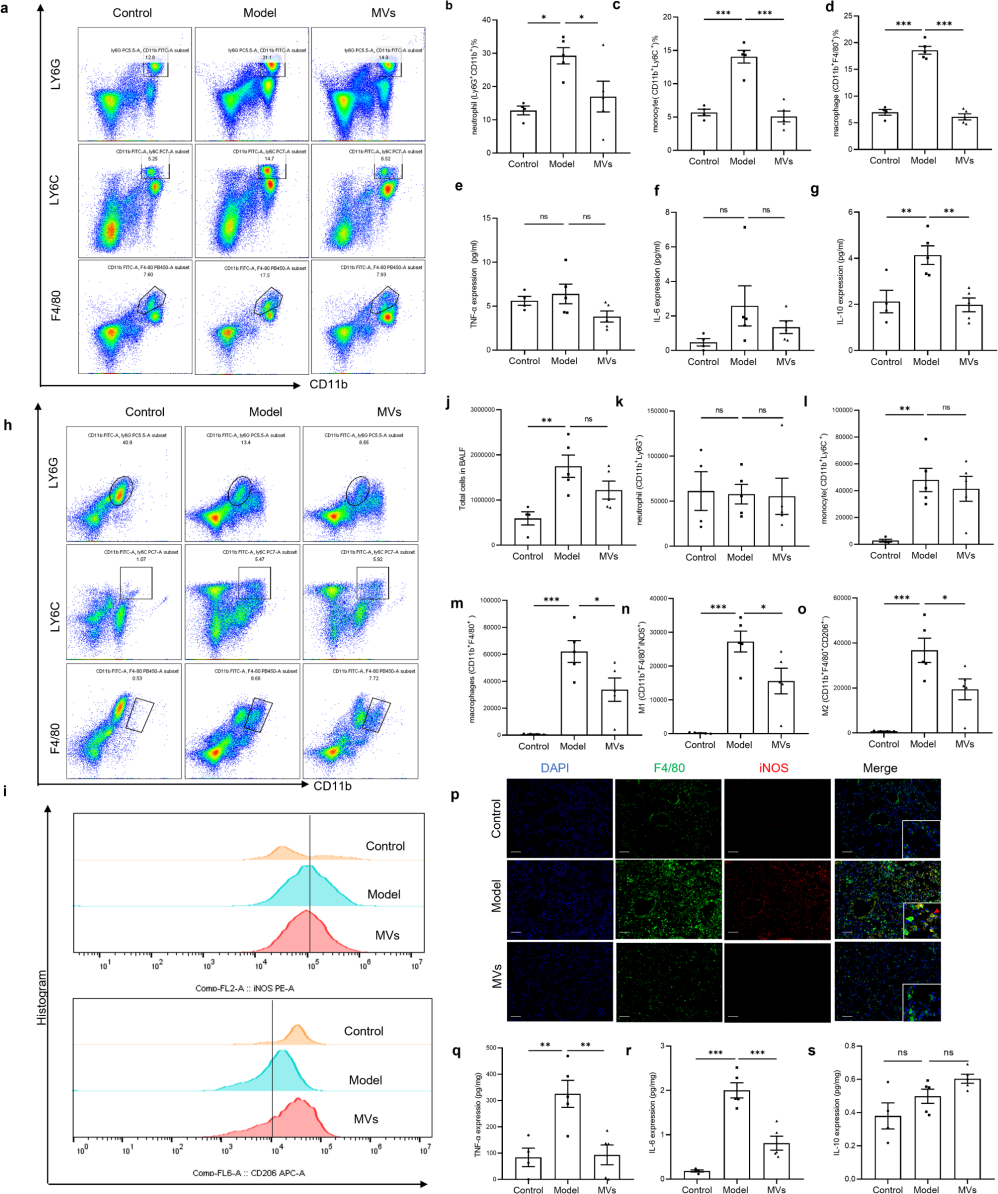
MSC-MVs inhibited monocytes and macrophages in early BLM-induced PF in mice. (**a**) Flow cytometric analysis of neutrophils, monocytes and macrophages in the peripheral blood of animals in the control, model, and MV groups. (**b**-**d**) Statistical analysis of the flow cytometric results of neutrophils and macrophages in the peripheral blood of the three groups. (**e**-**g**) The serum levels of TNF-α, IL-6, and IL10 in the three groups were measured. (**h**, **i**) Flow cytometric analysis of neutrophils, monocytes, macrophages, M1 macrophages and M2 macrophages in the BALF of the three groups. (**j**) Statistical analysis of the number of total cells in the BALF of the three groups. (**k**-**o**) Statistical analysis of the flow cytometric results for neutrophils, monocytes, macrophages, M1 macrophages and M2 macrophages in the BALF of the three groups. (**p**) IF assay showing the protein expression of F4/80 and iNOS in the lung tissue of the three groups. Scale bar = 100 μm. (**q**-**s**) CBD was used to measure the levels of TNF-α, IL-6, and IL-10 in the lung tissue homogenates of the three groups. *n* = 5. The data are shown as the means ± SEMs. The Shapiro-Wilk test of the data > 0.05. One-way ANOVA followed by the Sidak multiple comparison test was used to identify differences among the three groups, and ∗ indicates the difference between the control and model groups or the difference between the model group and the MV group. **p < 0.01. ***p < 0.001

In this study, we determined the expression levels of innate immune cells (neutrophils, monocytes and macrophages) in peripheral blood and BALF and the levels of the corresponding inflammatory cytokines on day 7 by flow cytometry (Fig. 3). Flow cytometric analysis of neutrophils, monocytes and macrophages in the peripheral blood of the animals in the control, model, and MSC-MV groups is shown in Fig. 3a. Compared with that in the control group, the percentage of neutrophils (CD11b^+^ Ly6G^+^) in the peripheral blood of the model group was greater, while the proportion of neutrophils decreased substantially after MSC-MV treatment (*p* < 0.05). Neutrophils and macrophages also showed similar changes (*p* < 0.05) (Fig. 3c and d). In addition, compared with those in the control group, the serum levels of inflammatory cytokines, including TNF-α and IL-6, in the model group were slightly greater, and these levels decreased slightly after MSC-MV treatment, but the differences were not statistically significant (Fig. 3e and f). However, compared with that in the control group, the serum level of IL-10 decreased in the model group and increased in the MV group, and the differences were statistically significant (*p* < 0.001) (Fig. 3g).

Furthermore, flow cytometric analysis of neutrophils, monocyte macrophages, M1 macrophages and M2 macrophages in the BALF of the three groups is shown (Fig. 3h and i). The results revealed that the number of total cells in the BALF increased in the model group (*p* < 0.05) but decreased slightly in the MV group (*p* > 0.05) (Fig. 3j). The number of neutrophils in the BALF did not significantly differ among the three groups (Fig. 3k). Compared with those in the control group, the number of monocytes in the model group increased (*p* < 0.05) but decreased slightly after MSC-MV treatment (*p* > 0.05). Neutrophils and macrophages also showed similar changes (*p* < 0.05) (Fig. 3l). Compared with those in the control group, the number of macrophages increased distinctly in the model group and decreased substantially after MSC-MV treatment, and similar changes were observed in the numbers of both M1 and M2 macrophages in the BALF of the three groups (Fig. 3m-o). Moreover, the IF results of lung tissues suggested that MSC-MVs can reduce total macrophage (F4/80^+^) and M1 cell (F4/80^+^iNOS^+^) infiltration in lung tissues (Fig. 3p). Our results revealed that the levels of TNF-α and IL-6 in the lung homogenates decreased in the model group and increased in the MV group (*p* < 0.001) (Fig. 3q and r), whereas the levels of IL-10 in the lung homogenates did not significantly differ among the three groups (Fig. 3s). In conclusion, MSC-MVs reduced the levels of monocytes, monocyte-derived macrophages and related inflammatory cytokines in the peripheral blood and lung tissue of mice with early PF.

### MSC-MVs inhibited chemotaxis via the CCL2/CCR2 axis to regulate the chemotaxis of monocytes and macrophages

**Fig. 4:**
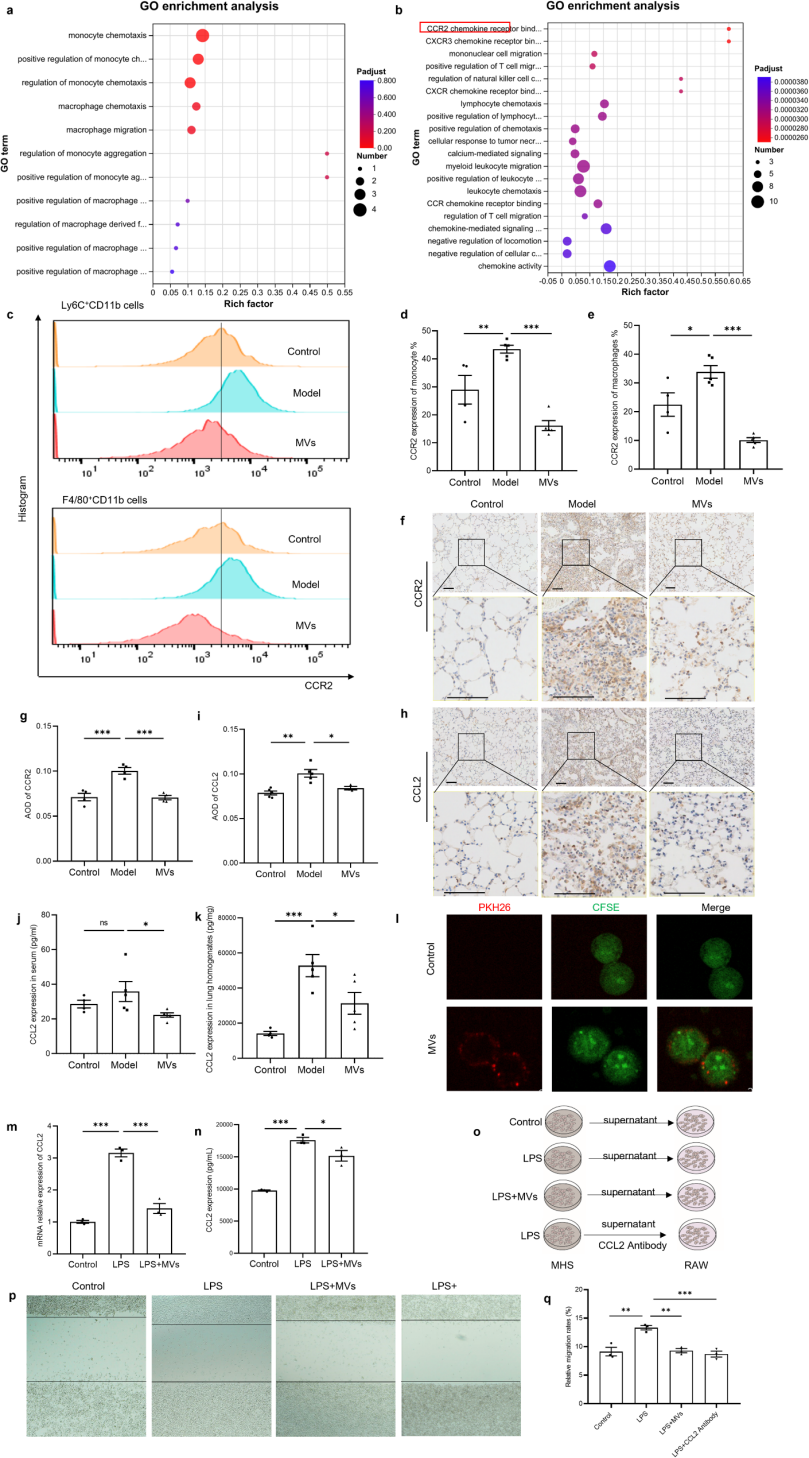
MSC-MVs inhibited chemotaxis through the CCL2/CCR2 axis to regulate the chemotaxis of monocytes and macrophages. (**a**) Bubble chart of the GO enrichment of 116 genes related to monocytes and macrophages. The color of the bubbles indicates the *p* value, and the size of the bubbles indicates the number of genes associated with the GO term. (**b**) Bubble chart of the GO enrichment of 28 genes related to the migration of monocytes and macrophages. The color of the bubbles indicates the *p* value, and the size of the bubbles indicates the number of genes associated with the GO term. (**c**) Flow cytometric analysis showing CCR2 expression in monocytes and macrophages in the peripheral blood of the control, model, and MV groups. (**d**, **e**) Statistical analysis of the flow cytometric results showing CCR2 expression in monocytes and macrophages in the peripheral blood of the three groups (*n* = 5). (**f**, **h**) Representative images of immunohistochemical staining for CCR2 and CCL2. (**g**, **i**) Comparative analysis of the AOD values showing positive staining for CCR2 and CCL2 in lung tissues (*n* = 5). (**j**) Serum CCL2 protein levels in the three groups(*n* = 5). (**k**) CCL2 levels in the lung homogenates of the three groups(*n* = 5). (**l**) Confocal microscopy revealed that MHS cells take up MSCs-MVs. MHS cells were stained with CFSE (green), and MSC-MVs were stained with PKH26 (red). (**m**) CCL2 mRNA levels in MHS cells in the control, LPS and LPS + MV groups (*n* = 3). (**n**) The protein level of CCL2 in the MHS cell supernatants of the control, LPS and LPS + MV groups(*n* = 3). (**o**) Experimental program for assessing the migration of RAW264.7 cells. (**p**) Representative images of the wound-healing assay of RAW264.7 cells in the three groups. (**q**) Comparative analysis of relative migration rates (%) in the three groups(*n* = 3). The data are shown as the means ± SEMs. The Shapiro-Wilk test of the data > 0.05. One-way ANOVA followed by the Sidak multiple comparison test was used to identify differences among the three groups, and * indicates a difference between the control and model groups or between the model group and the MV group. **p < 0.01. ***p < 0.001

Further, the GO functions of monocytes and macrophages about 116 distinct genes focus on migration and chemotaxis (Fig. 4a). Then, genes related to monocyte and macrophage chemotaxis were screened, and 28 different genes, such as CCL2, CCL3 and SPP1, were identified. GO functional enrichment analysis of these 28 genes revealed that “CCR2 chemokine receptor binding”, “CXCR3 chemokine receptor binding”, and other categories were enriched (Fig. 4b). The CCL2/CCR2 chemotactic axis is the classic chemotactic pathway for monocytes and macrophages [25, 26]. Hence, we first determined the levels of CCL2 and CCR2 in the peripheral blood and lung tissue of the mice in the three groups.

Flow cytometric analysis of peripheral blood revealed that, compared with those in the control group, the percentages of CCR2-positive monocytes and macrophages in the model group were greater, whereas the proportions of these cells decreased substantially after MSC-MV treatment (*p* < 0.05) (Fig. 4c-e). Immunohistochemical results revealed that the protein expression of both CCR2 and CCL2 was significantly greater in the model group than in the control group and was significantly lower in the MSC-MV group than in the model group (Fig. 4f-i). In addition, the levels of CCL2 in both the serum and lung homogenates increased in the model group but decreased after MSC-MV treatment (Fig. 4j and k). These findings suggested that MSC-MVs inhibit the CCL2/CCR2 chemotactic axis in vivo.

Activated macrophages and alveolar epithelial cells in lung tissue are the main cell types responsible for the production of CCL2 [27, 28]; therefore, MHS cells were used to further verify the role of MSC-MVs in this chemotactic axis. First, MHS cells were stained with the green fluorescent dye CFSE, and MSC-MVs were stained with the red fluorescent dye. After the MHS cells were cocultured with the MSC-MVs for 3 h, confocal microscopy revealed the entry of the MSC-MVs into the MHS cells (Fig. 4l). Furthermore, we observed an increase in the CCL2 mRNA and protein levels in MHS cells following stimulation with LPS. Interestingly, coculture with MSC-MVs partially reversed this altered expression pattern (Fig. 4m and n), and then pre-experiment suggested the effects of MSC-MVs exhibit a dose-dependent manner for regulation of CCL2 expression (Supplementary Fig. 5).

Finally, we obtained supernatants from the MHS cells in the control, LPS and LPS + MSC-MV groups to coculture with RAW264.7 cells, mouse monocyte macrophages (Fig. 4o). A scratch assay revealed that LPS-treated MHS cells promoted RAW264.7 cell migration, whereas MSC-MVs and CCL2 antibody inhibited this effect (Fig. 4p and q). These findings suggest that MSC-MVs may inhibit the migration of monocytes and macrophages by suppressing the CCL2/CCR2 signaling pathway.

### MSC-MVs induced ERK1/2 protein phosphorylation to inhibit the expression of CCL2 in vivo and in vitro

**Fig. 5:**
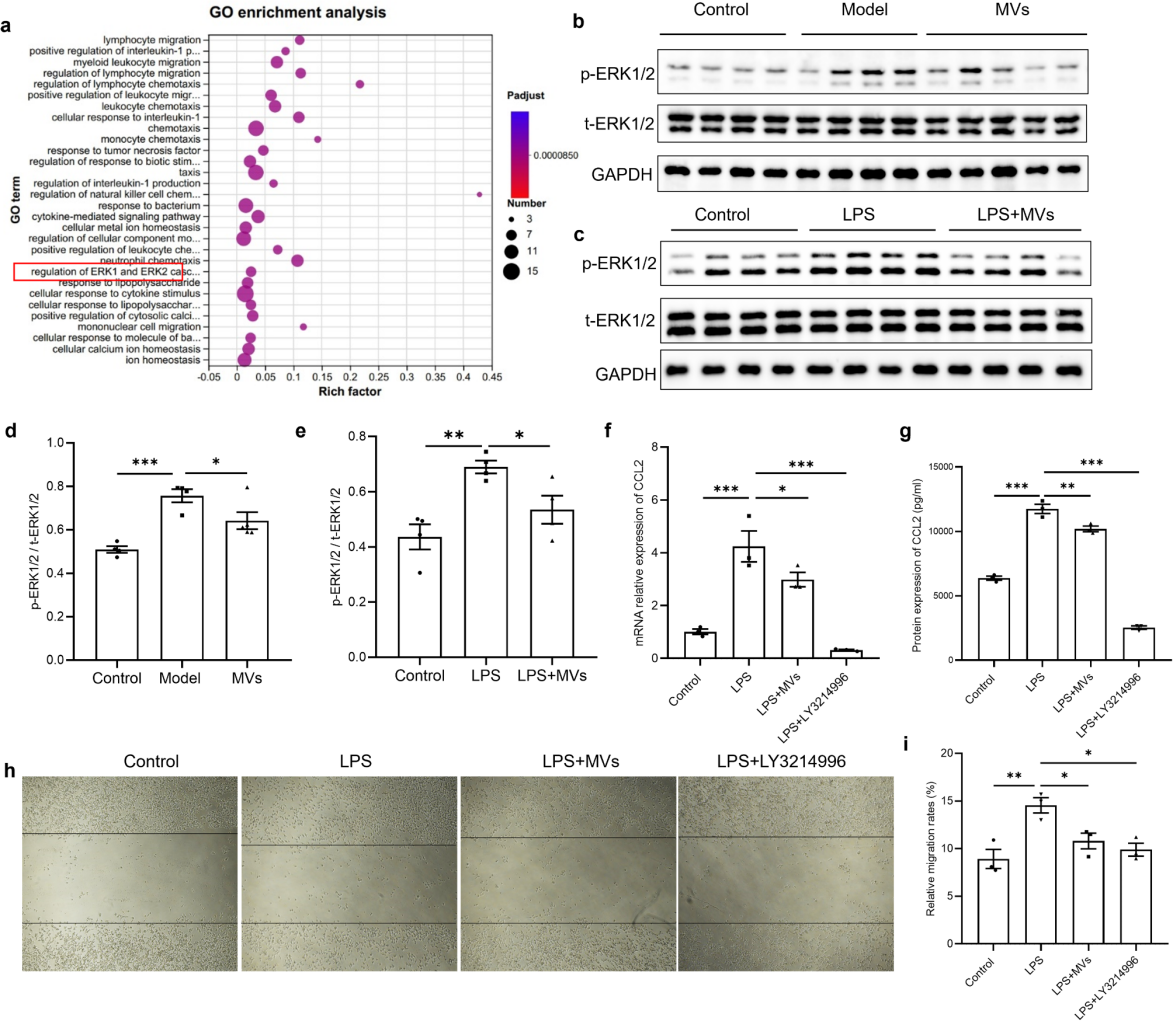
MSC-MVs induced ERK1/2 protein phosphorylation to inhibit CCL2 expression. (**a**) Bubble chart of the GO enrichment of 62 genes related to CCL2. The color of the bubbles indicates the *p* value, and the size of the bubbles indicates the number of genes associated with the GO term. (**b**, **d**) The corresponding levels of phosphorylated ERK1/2 (p-ERK1/2) and total ERK1/2 (t-ERK1/2) in the lung tissues of mice in the control, model, and MV groups were determined by WB, with GAPDH as an internal reference. The corresponding uncropped full-length blots are included in Supplementary Fig. 2b (*n* = 4–5). (**c**, **e**) The corresponding levels of p-ERK1/2 and t-ERK1/2 in the MHS cells in the control, LPS and LPS + MV groups were determined by WB with GAPDH as an internal reference. The corresponding uncropped full-length blots are included in Supplementary Fig. 2c (*n* = 4). (**f**, **g**) The mRNA and protein levels of CCL2 in MHS cells in the control, LPS, LPS + MV and LPS + LY3214996 groups (*n* = 3). (**H**) Representative images of the wound-healing assay of RAW264.7 cells in the four groups. (**i**) Comparative analysis of relative migration rates (%) in the four groups (*n* = 3). The data are shown as the means ± SEMs. The Shapiro-Wilk test of the data > 0.05. One-way ANOVA followed by the Sidak multiple comparison test was used to identify differences among the three groups, and * indicates the difference between the control and model groups or the difference between the model group and the MV group. **p < 0.01. ***p < 0.001

Furthermore, we performed transcriptomic analysis to investigate the mechanism underlying the regulation of CCL2 by MSC-MVs. According to the RNA-seq data, 62 different genes were found to encode CCL2. GO functional enrichment analysis of these genes identified the ERK1/2 pathway (Fig. 5a). Therefore, the levels of phosphorylated ERK1/2 (p-ERK1/2) and total ERK1/2 (t-ERK1/2) in vivo were determined via WB, and the results revealed that the expression level of p-ERK1/2 increased in the model group but decreased after MSC-MV treatment (Fig. 5b and d). Furthermore, MSC-MVs decreased the increase in p-ERK1/2 in MHS cells stimulated with LPS (Fig. 5c and e). Finally, using LY3214996, an inhibitor of the ERK1/2 pathway, we examined the expression of CCL2 and the migration of RAW264.7 cells and found that LY3214996 decreased the increase in the CCL2 level in the MHS cells stimulated with LPS and inhibited the migration of RAW264.7 cells, which indicated that MSC-MVs inhibited the expression of CCL2 and the migration of RAW264.7 cells by inducing ERK1/2 protein phosphorylation (Fig. 5f-i).

## Discussion

**Fig. 6:**
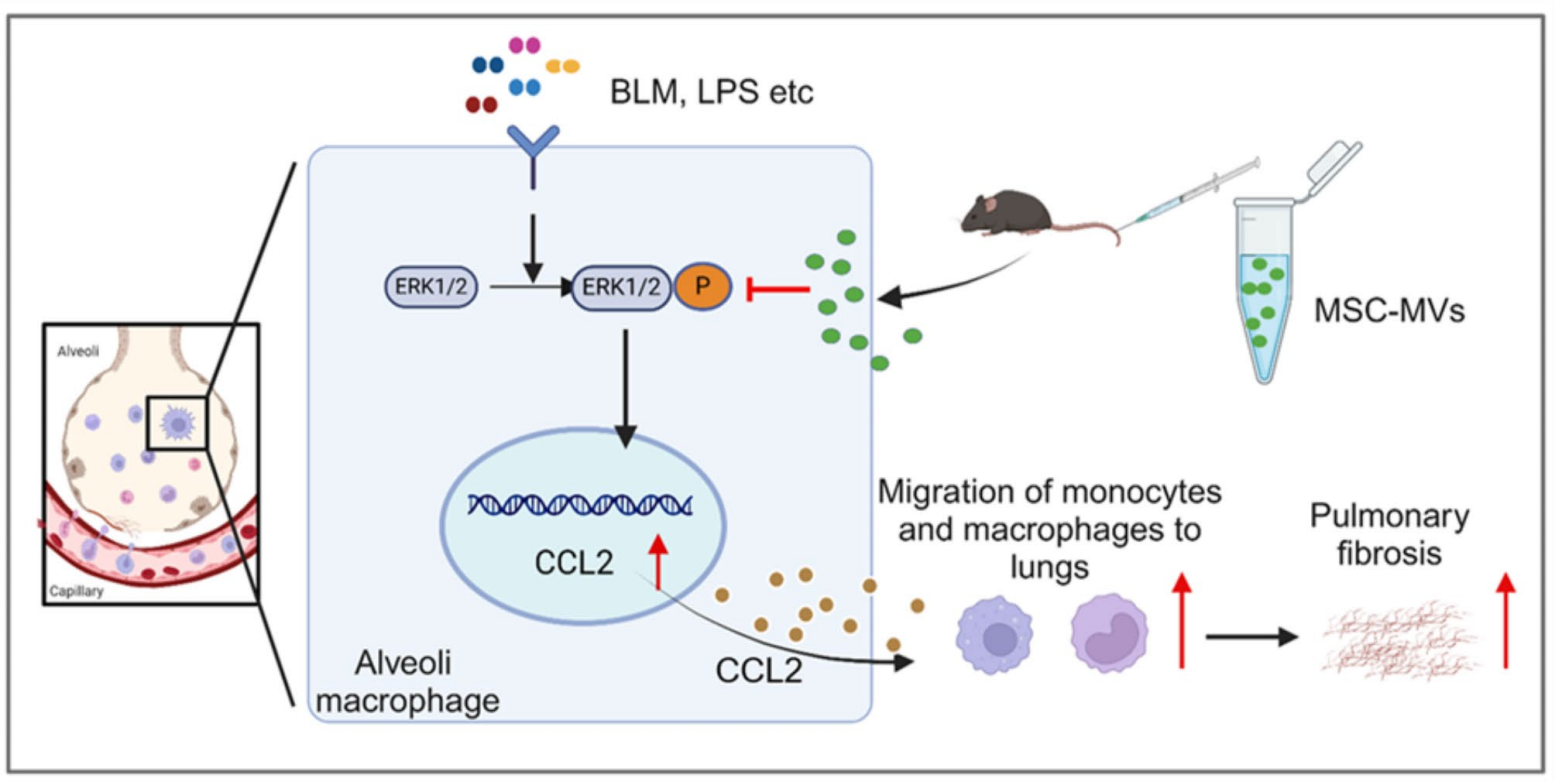
Diagram of the mechanism. In lung tissues affected by PF, the ERK1/2 protein is phosphorylated in activated AMs, leading to abundant CCL2 production and release. CCL2 binds to CCR2 on monocytes and macrophages and then recruits these cells to lung tissue, which promotes the progression of PF. MSC-MVs blocked the phosphorylation of ERK1/2 to inhibit this process

PF is a collective term for a group of lung diseases characterized by persistent inflammation and progressive fibrosis, which commonly occur because of various pulmonary conditions [2, 7, 29]. In recent years, stem cell therapy has emerged as a prominent area of research, showing promise for the treatment of PF [30–33]. In this study, we demonstrated the effective inhibitory effects of MSC-MVs on BLM-induced PF. Our findings revealed that MSC-MVs exerted regulatory effects on monocytes, monocyte-derived macrophages and associated inflammatory cytokines in peripheral blood and lung tissue. Notably, treatment with MSC-MVs significantly attenuated the monocyte‒macrophage chemotactic CCL2/CCR2 axis. This notable effect was supported by lung transcriptomic data as well as in vitro and in vivo validation experiments, which confirmed that MSC-MVs hindered CCL2 production through the inhibition of ERK protein phosphorylation (Fig. 6).

An uncontrolled immune response and an imbalanced damage-inflammation-repair process drive the development and progression of PF. Immune cells from both the innate and adaptive immune systems, including granulocytes, monocytes, macrophages, dendritic cells, regulatory T cells (Tregs), regulatory B cells (Bregs), and macrophages, play a role in the pathogenesis of PF, especially monocyte-derived macrophages [34]. Neutrophils may play a role in the development of PF by releasing neutrophil extracellular traps and secreting neutrophil elastase [35]. In addition, neutrophils release various MMPs that are involved in the deposition and maintenance of the extracellular matrix [9]. CXCL4 can modulate the development of dendritic cells toward a proinflammatory and profibrotic phenotype, consequently leading to extracellular matrix accumulation and myofibroblast transformation [36]. The numbers of Tregs and Bregs in the peripheral blood and BALF of IPF patients are reduced, which is correlated with the severity of the disease [37]. Macrophages promote the development of PF by releasing soluble factors such as IL-1β, IL-6, TNF-α, TGF-β, and MMP and extracellular matrix remodeling [38, 39]. Furthermore, macrophages contribute to fibrosis by engulfing apoptotic cells through exocytosis, which then activates TGF-β [9]. In this study, we found that MSC-MVs reduced the levels of innate immune cells, including granulocytes, monocytes and macrophages, in mice with BLM-induced PF. The regulatory effect of MSCs-MVs on macrophages was particularly prominent in our study, so we next explored the specific mechanism involved.

The macrophages found in lung tissue come from two different sources: resident macrophages are in the alveoli and interstitium, and monocytes are recruited to damaged lung tissue under inflammatory conditions [12]. When these monocytes migrate into the tissue, they transform into dormant M0-type macrophages, which can then polarize into either classically activated M1 macrophages or selectively activated M2 macrophages in response to various inflammatory stimuli [40]. Current evidence indicates that both M1 and M2 macrophages play a role in promoting PF [10]. M1 macrophages are central mediators of the inflammatory phase of PF and release proinflammatory cytokines and chemokines such as IL-1β, IL-6, IL-12, IL-23, CCL2 and TNF-α [41–43]. M2 macrophages can produce profibrotic factors such as TGF-β and PDGF, which can trigger prolonged activation of fibroblasts and promote the proliferation of myofibroblasts [44]. Alexander et al. [13] reported that intratracheal administration of liposomal clodronate to deplete resident macrophages has no effect on PF, whereas elimination of monocyte-derived macrophages expressing CD11b may help alleviate fibrosis [45], suggesting that the primary source of macrophages responsible for PF is monocytes. Therefore, repressing the activity of macrophages is an important treatment objective for pulmonary interstitial fibrosis, especially for inhibiting monocyte-derived macrophages. In recent years, investigations into the therapeutic potential of MSC-EVs have revealed that MSC-EVs can modulate macrophage phenotypic changes, thereby ameliorating diseases such as cardiovascular, digestive, renal and central nervous system disorders [46]. For pulmonary diseases, Willis et al. [47] reported both in vivo and in vitro that UCMSC-EXOs effectively regulate macrophage phenotypes, suppressing proinflammatory M1 states (CCL2, CCL7, and IL-6) while promoting anti-inflammatory M2-like states (arginase-1, CD206, and CCL17), and these findings demonstrate the potential of UCMSC-EXOs to alleviate bronchopulmonary dysplasia induced by hyperoxia by normalizing lung function, reducing fibrosis and pulmonary vascular remodeling, and ameliorating pulmonary hypertension. Similarly, Mansouri et al. [23] reported that the administration of BMSC-EXOs reduced the number of classic proinflammatory monocytes (CD45^+^CD11b^+^MHCII^−^CD64^lo/int^CCR2^+^Ly6C^chi^) while simultaneously increasing the levels of nonclassic monocytes (CD45^+^CD11b^+^MHCII^−^CD64^lo/int^CCR2^−^Ly6C^lo)^ and AMs (CD45^+^ CD11b^−^CD11c^+^CD64^+^) to mitigate BLM-induced PF. In our study, we observed that MSC-MVs suppressed monocytes and monocyte-derived macrophages (including M1 and M2 macrophages) to ameliorate PF in BLM-induced mice.

Monocytes and monocyte-derived macrophages are recruited mainly to lung tissue through the CCL2/CCR2 chemotaxis pathway. Deletion of the CCR2 gene or the use of CCL2 neutralizing antibodies significantly reduced the number of macrophages in lung tissue, alleviating PF [48, 49]. Our findings showed that MSC-MVs reduced the level of CCL2 in lung tissues and peripheral blood and downregulated CCR2 expression in monocytes and monocyte-derived macrophages, subsequently impeding their migration from peripheral blood to lung tissues. Many cell types, including endothelial cells, fibroblasts, epithelial cells, smooth muscle cells, mesangial cells, astrocytes, monocytes, and microglia, have been identified as producers of CCL2 [50]. Notably, activated macrophages and alveolar epithelial cells in lung tissue are recognized as the primary sources of chemokines such as CCL2, highlighting their crucial role in mediating immune responses within this specific organ [27, 28]. Lei et al. [51] reported that type II alveolar epithelial cells produce CCL2 under flagellin stimulation and then recruit monocytes to the lungs, where they differentiate into macrophages. Nie et al. [52] reported that an increase in the production of CCL2 and CXCL2 in macrophages can promote PF. In our study, we also found that activated macrophages produced more CCL2 and that MSC-MVs inhibited this process. In activated macrophages, the ERK pathway is promoted and mediates their biological functions [53–55]. Furthermore, lung transcriptomic data suggested the involvement of the ERK1/2 pathway in this process, and this marked effect was supported by in vitro and in vivo validation experiments. Overall, our findings confirmed that MSC-MVs can effectively suppress CCL2 production in activated AMs by inhibiting the phosphorylation of ERK1/2.

In summary, compared with MSCs, MVs represent an innovative cell-free treatment modality for PF. However, our study has certain limitations that need to be addressed. First, while the dose of MVs was calculated based on the number of particles in most of the literature, its representation of the active component of MVs remains unclear. Additionally, relying solely on protein concentration poses challenges. Second, this study focused primarily on evaluating the efficacy of a single injection of MVs; however, future studies should include dose‒response experiments to evaluate the clinical application of these agents. Furthermore, researchers should further explore alternative administration routes and compare nebulization with intravenous administration to determine whether local delivery still promotes alleviation of BLM-induced PF.

## Conclusions

In conclusion, our findings demonstrated the potent inhibitory effect of MSC-MVs on BLM-induced PF, including robust inhibition of pulmonary inflammation, collagen deposition, and the expression of key extracellular matrix components. Furthermore, our results revealed that MSC-MVs effectively suppressed the migration of monocytes and macrophages by suppressing CCL2 expression through the modulation of the ERK1/2 signaling pathway, thereby alleviating PF. These findings provide a solid theoretical foundation for understanding the pivotal role of MSC-MVs in the pathogenesis of PF.

## Electronic supplementary material

Below is the link to the electronic supplementary material.

Supplementary Material 1

## Data Availability

The fastq files were deposited into the NCBI Sequence Read Archive (SRA) database (accession number: PRJNA1118833).

## Abbreviations

PF: Pulmonary fibrosis
CTD: Connective tissue disease
ELISA: Enzyme-linked immunosorbent assay
RT‒qPCR: Real-time quantitative polymerase chain reaction
AMs: Alveolar macrophages
MSCs: (Human umbilical cord) mesenchymal stem cells
EVs: Extracellular vesicles
EXOs: Exosomes
MVs: Microvesicles
BLM: Bleomycin
PBS: Phosphate-buffered saline
TEM: Transmission electron microscopy
NTA: Nanoparticle tracking analysis
WB: Western blotting
ERK: Extracellular regulated protein kinases
GAPDH: Glyceraldehyde-phosphate dehydrogenase
HE: Hematoxylin and eosin
TBS: Tris-buffered saline
DAB: Diaminobenzidine
CCR2: C-C motif chemokine receptor 2
CCL2: C-C motif chemokine ligand 2
GO: Gene ontology
BP: Biological process
CC: Cellular component
MF: Molecular function
MHS: Murine alveolar macrophage line
FBS: Fetal bovine serum
LPS: Lipopolysaccharide
BALF: Bronchoalveolar lavage fluid
AOD: Average optical density
