# Supplementary material for: Human umbilical cord mesenchymal stem cell-derived microvesicles alleviate pulmonary fibrosis by inhibiting monocyte‒macrophage migration through ERK1/2 signaling-mediated suppression of CCL2 expression
Source: Stem Cell Res Ther. 2025 Mar 24;16:145. doi: 10.1186/s13287-025-04266-w (PMC11934500; doi:10.1186/s13287-025-04266-w)

Supplementary Table 1 Primer sequences

| Primer name | Primer sequence | |
| --- | --- | --- |
| mCCL2 | forward | 5’- GCTACAAGAGGATCACCAGCAG-3’ |
|  | reverse | 5’- GTCTGGACCCATTCCTTCTTGG-3’ |
| mGAPDH | forward | 5’- CATCACTGCCACCCAGAAGACTG-3’ |
|  | reverse | 5’- ATGCCAGTGAGCTTCCCGTTCAG-3’ |

Abbreviations: CCL2: The C-C motif chemokine ligand 2; GAPDH: glyceraldehyde-phosphate dehydrogenase.

| Antibody name | Product number | Company | Dilution |
| --- | --- | --- | --- |
| anti-CCR2 | GB11326 | servicebio | IHC: 1:800 |
| anti-CCL2 | GB11199 | servicebio | IHC: 1:500 |
| anti-Collagen Ⅰ | ab138492 | Abcam | IHC: 1:1000  WB: 1:2000 |
| anti-α-SMA | ab7817 | Abcam | IHC: 1:1000  WB: 1:2000 |
| anti-FN | ab2413 | Abcam | IHC: 1:500  WB: 1:5000 |
| anti-F4/80 | GB113373/ | servicebio | IF: 1:2000 |
| anti-INOS | GB11119 | servicebio | IF: 1:100 |
| anti-CD9 | abcam | ab92726 | WB: 1:2000 |
| anti-CD163 | ABSIN | ABS134386 | WB: 1:2000 |
| anti-TSG101 | servicebio | GB11618 | WB: 1:2000 |
| anti-ERK1/2 | 4695S | Cell Signaling | WB: 1:2000 |
| anti-P-ERK1/2 | 4370S | Cell Signaling | WB: 1:2000 |
| anti-GAPDH | TA802519 | OriGene | WB: 1:2000 |

Supplementary Table 2 WB and HIC antibody details

Supplementary Table 3 Flow antibody details

| Antibody name | Conjugation | Company | product number | Dilution |
| --- | --- | --- | --- | --- |
| anti-ly6G | PerCP-Cy 5.5 | BD | 560602 | 1:400 |
| anti-CD11b | FITC | BD | 553310 | 1:400 |
| anti-Ly6C | PE-Cy7 | BD | 580593 | 1:400 |
| anti-F4/80 | BV421 | BD | 565411 | 1:200 |
| anti-CD206 | APC | BD | 565250 | 1:200 |
| anti-iNOS | PE | eBioscience | 2151410 | 1:200 |
| anti-CCR2 | PE | BioLegend | 150609 | 1:200 |


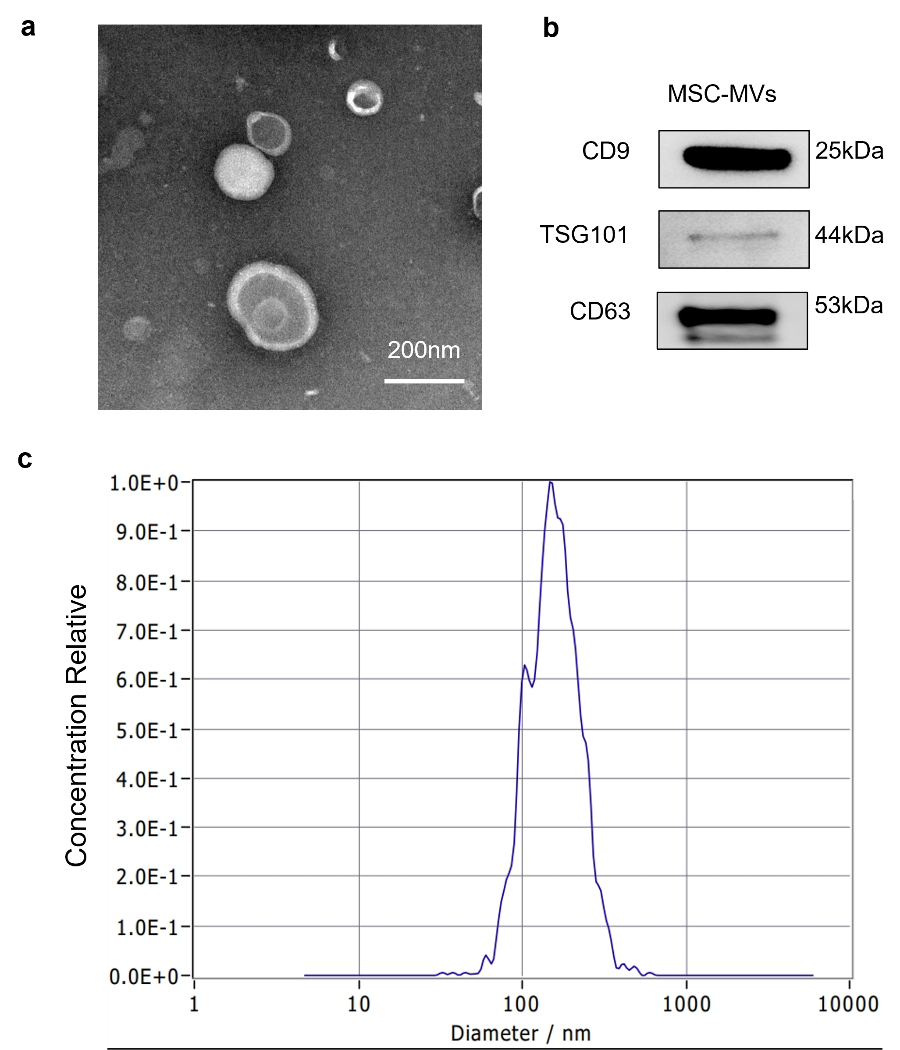


Supplementary Figure 1: Characteristics of MSC-MVs. (a) A cup-shaped morphology of MSC-MVs was shown by TEM, scale bar 200 nm; (b) MSC-MVs expressed CD9, CD63 and TSG101 proteins and the corresponding uncropped full-length blot were included in the supplementary figure 2A. (c) Nanosight analysis of MSC-MVs showed a single peak at 100 nm. Abbreviations: MSC-MVs: microvesicles derived human umbilical cord mesenchymal stem cells; TEM: Transmission electron microscopy. Abbreviation: MSC-MVs: human umbilical cord mesenchymal stem cells microvesicles; TEM: Transmission electron microscopy; NTA: Nanoparticle tracking analysis.


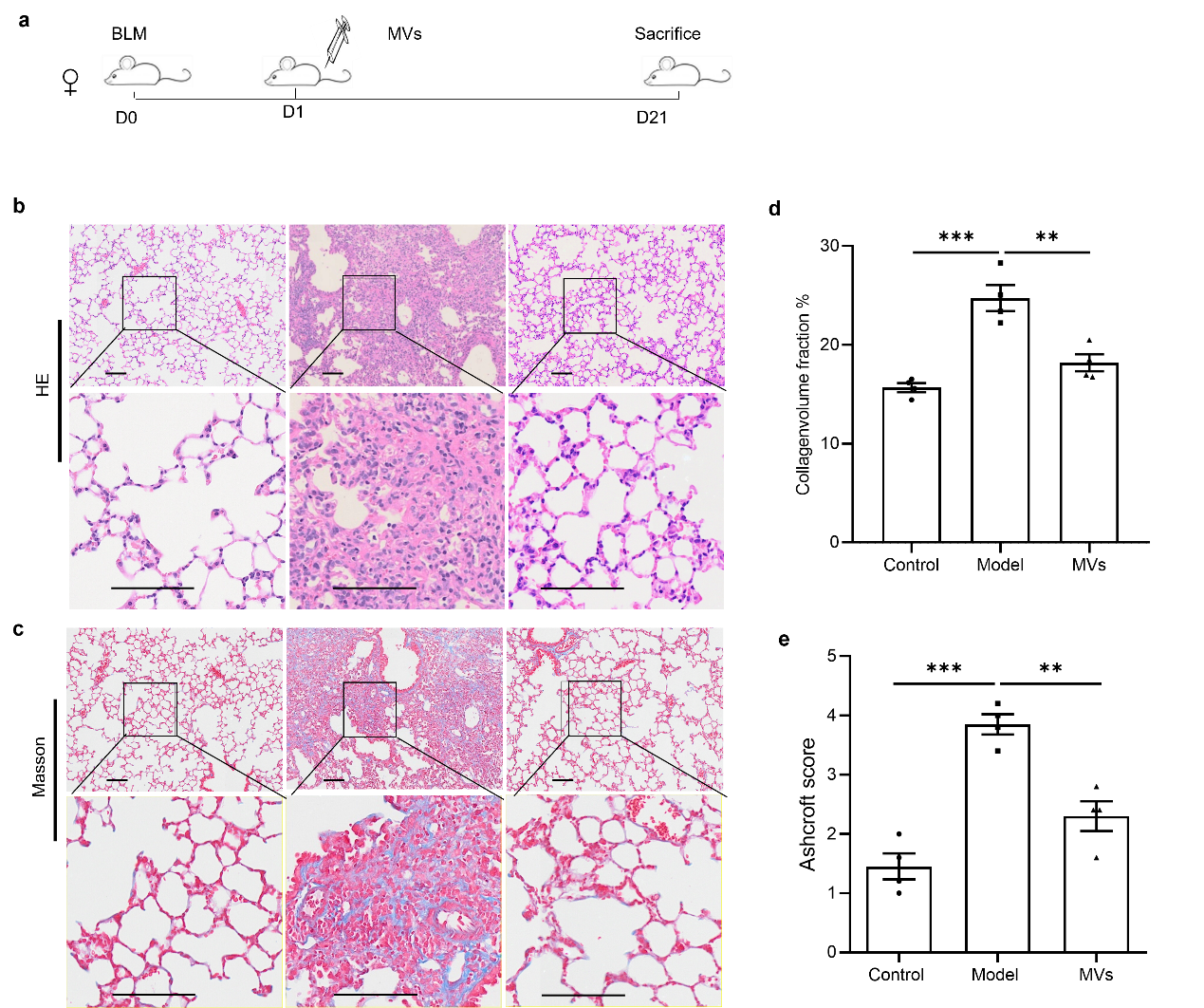


Supplementary Figure 2. Effects of MSC-MVs on BLM-induced PF in female mice (n=4). (a) Diagram of the experimental scheme. (b) HE staining of mouse lung tissue from the five groups. (c, d) Masson staining of mouse lung tissues from the five groups of mice and the statistical analysis of collagen-volume fraction %. (e) Ashcroft score of five groups. The data are shown as the means ± SEMs. The Shapiro-Wilk test of the data >0.05. One-way ANOVA followed by the Sidak multiple comparison test was used to identify differences among the three groups, and ∗ indicates the difference between the control and model groups or the difference between the model group and the MV group. ∗∗p <0.01. *** p<0.001. Scale bar, 100 μm. Abbreviations: SEM: standard error of the mean.


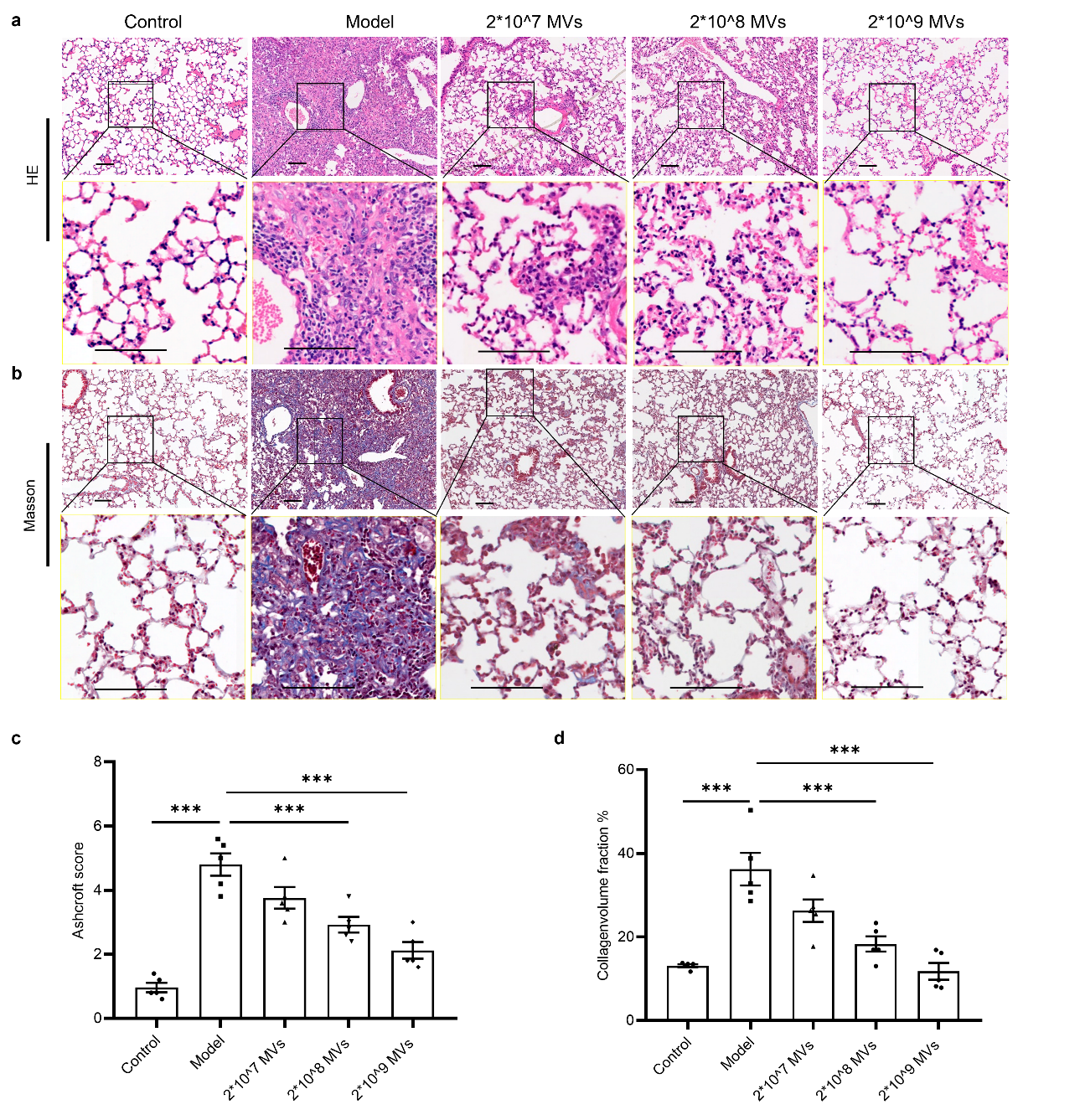


Supplementary Figure 3. Effects of different does MSC-MVs on BLM-induced PF in mice (n=5). (a) HE staining of mouse lung tissue from the five groups. (c) Ashcroft score of five groups. (b, d) Masson staining of mouse lung tissues from the five groups of mice and the statistical analysis of collagen-volume fraction %. The data are shown as the means ± SEMs. The Shapiro-Wilk test of the data >0.05. One-way ANOVA followed by the Sidak multiple comparison test was used to identify differences among the three groups, and ∗ indicates the difference between the control and model groups or the difference between the model group and the MV group. ∗∗p <0.01. *** p<0.001. Scale bar, 100 μm. Abbreviations: SEM: standard error of the mean.


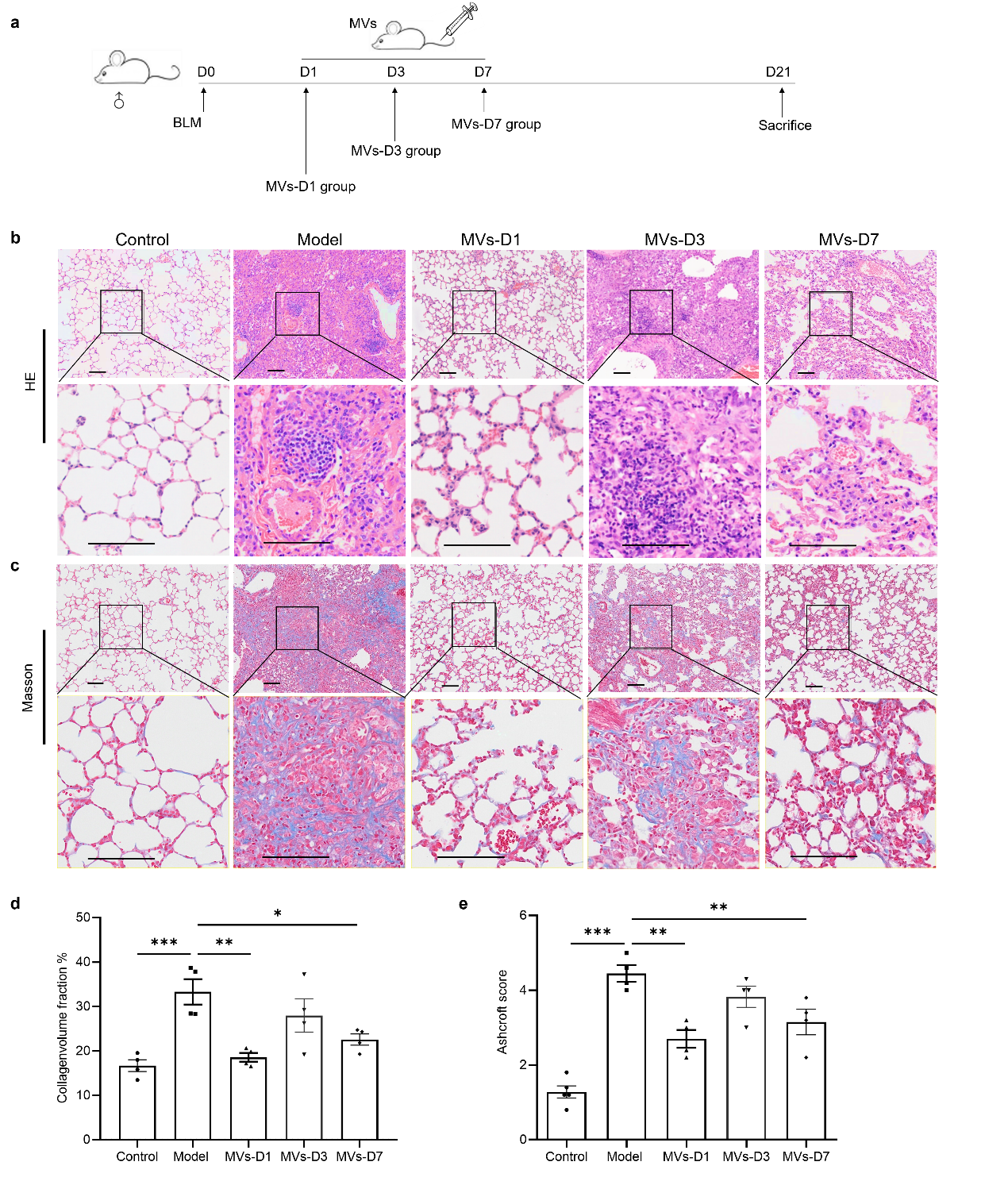


Supplementary Figure 4. Effects of administering same dose MSC-MVs to mice respectively on day1, 3, 7 post bleomycin (n=4). (a) Diagram of the experimental scheme. (b) HE staining of mouse lung tissue from the five groups. (c, d) Masson staining of mouse lung tissues from the five groups of mice and the statistical analysis of collagen-volume fraction %. (e) Ashcroft score of five groups. The data are shown as the means ± SEMs. The Shapiro-Wilk test of the data >0.05. One-way ANOVA followed by the Sidak multiple comparison test was used to identify differences among the three groups, and ∗ indicates the difference between the control and model groups or the difference between the model group and the MV group. ∗∗p <0.01. *** p<0.001. Scale bar, 100 μm. Abbreviations: SEM: standard error of the mean.


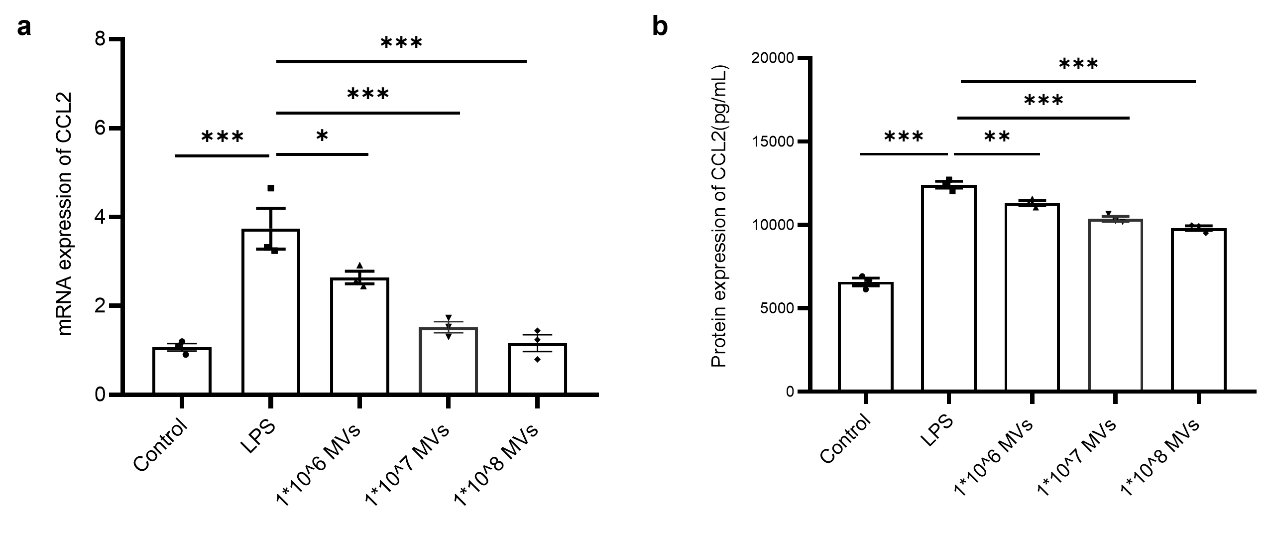


Supplementary Figure 5 (a) CCL2 mRNA levels in MHS cells in five groups (n=3). (b) The protein level of CCL2 in the MHS cell supernatants of five groups (n=3)


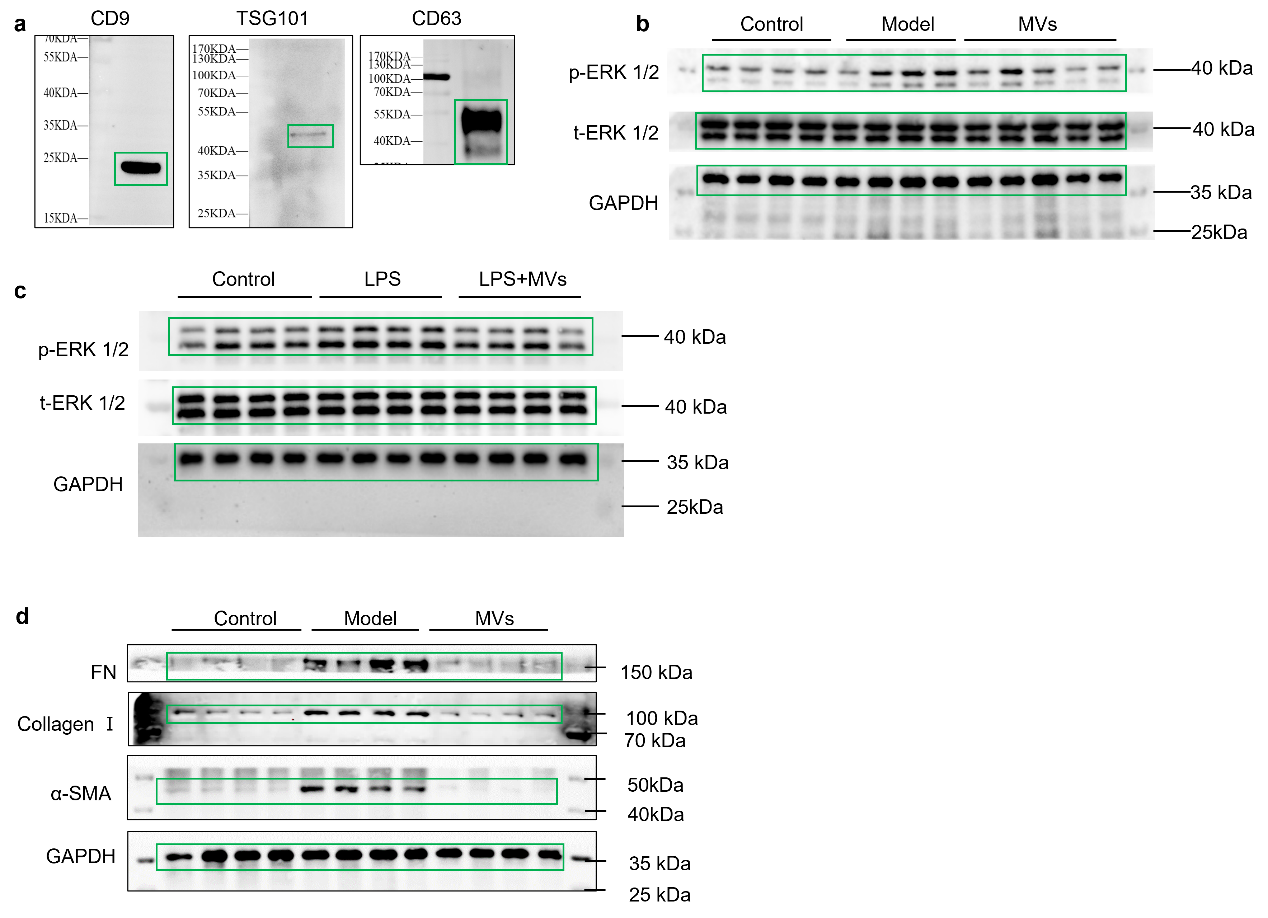


Supplementary Figure 6: Corresponding uncropped full-length blot. The green boxes were cropping marks.

Supplementary Figure 7: Heatmap across different groups for 116 different genes. The heatmap colors indicate normalized gene expression ranging from high (red) to low (blue).


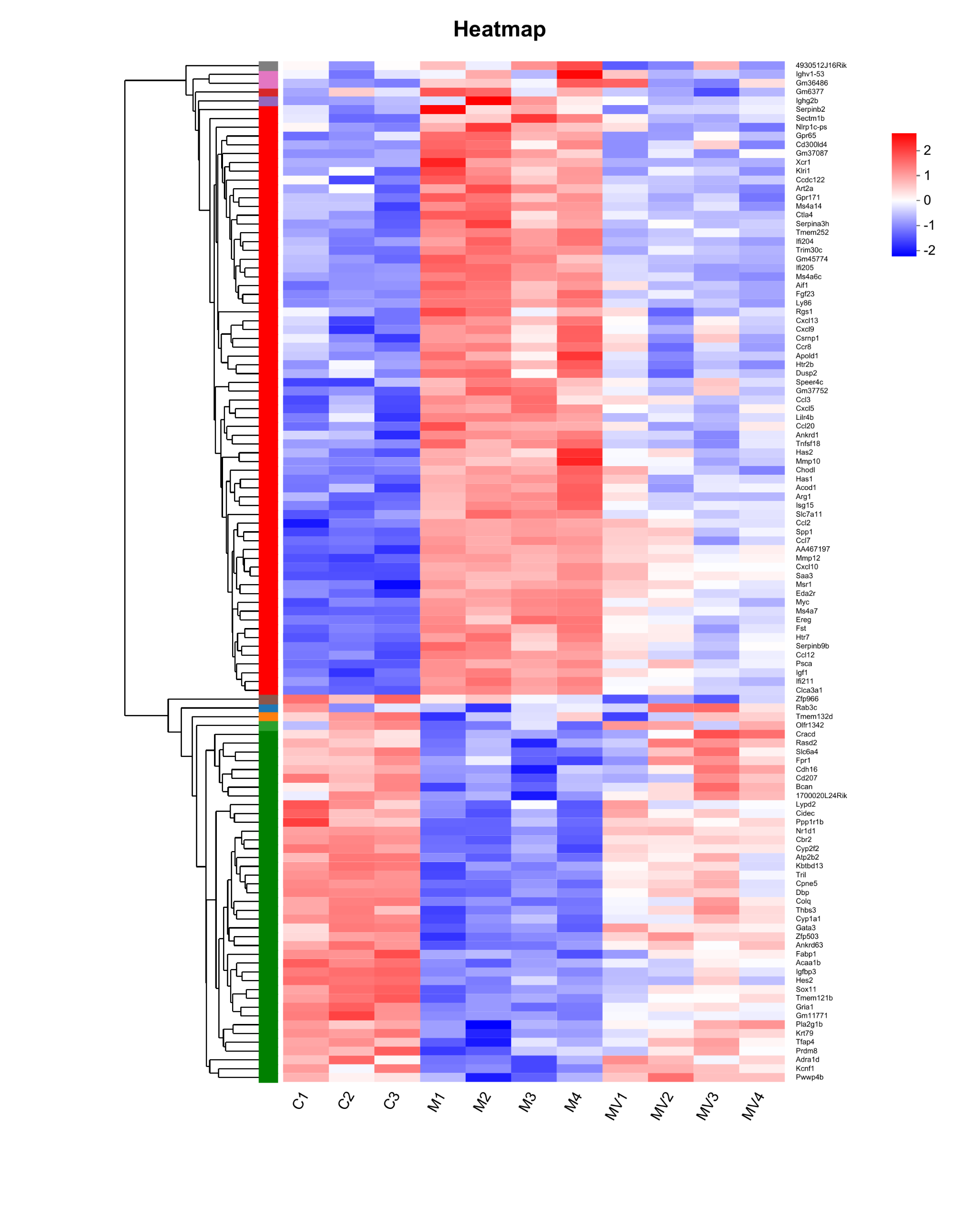

Supplement: Supplementary file 1 — Supplementary Material 1 [file 13287_2025_4266_MOESM1_ESM.docx]
